# A translational triage research development tool: standardizing prehospital triage decision-making systems in mass casualty incidents

**DOI:** 10.1186/s13049-021-00932-z

**Published:** 2021-08-17

**Authors:** Amir Khorram-Manesh, Johan Nordling, Eric Carlström, Krzysztof Goniewicz, Roberto Faccincani, Frederick M. Burkle

**Affiliations:** 1 Institute of Clinical Sciences, Department of Surgery, Sahlgrenska Academy, Gothenburg University, 413 45 Gothenburg, Sweden; 2 Gothenburg Emergency Medicine Research Group (GEMREG), Sahlgrenska Academy, 413 45 Gothenburg, Sweden; 3 Department of Research and Development, Armed Forces Center for Defense Medicine, 426 76 Västra Frölunda, Gothenburg, Sweden; 4 Institute of Health and Care Sciences, Sahlgrenska Academy, University of Gothenburg, 413 45 Gothenburg, Sweden; 5 USN School of Business, University of South-Eastern Norway, 3616 Kongsberg, Norway; 6 Department of Aviation Security, Military University of Aviation, 08-521 Dęblin, Poland; 7 Emergency Department, Humanitas Mater Domini, 210 53 Castellanza, Italy; 8 T.H. Chan School of Public Health, Harvard Humanitarian Initiative, Harvard University, Boston, MA 02115 USA

**Keywords:** Mass casualty incident, Prehospital, Tool, Trauma, Triage

## Abstract

**Background:**

There is no global consensus on the use of prehospital triage system in mass casualty incidents. The purpose of this study was to evaluate the most commonly used pre-existing prehospital triage systems for the possibility of creating one universal translational triage tool.

**Methods:**

The Rapid Evidence Review consisted of (1) a systematic literature review (2) merging and content analysis of the studies focusing on similarities and differences between systems and (3) development of a universal system.

**Results:**

There were 17 triage systems described in 31 eligible articles out of 797 identified initially. Seven of the systems met the predesignated criteria and were selected for further analysis. The criteria from the final seven systems were compiled, translated and counted for in means of 1/7’s. As a product, a universal system was created of the majority criteria.

**Conclusions:**

This study does not create a new triage system itself but rather identifies the possibility to convert various prehospital triage systems into one by using a triage translational tool. Future research should examine the tool and its different decision-making steps either by using simulations or by experts’ evaluation to ensure its feasibility in terms of speed, continuity, simplicity, sensitivity and specificity, before final evaluation at prehospital level.

**Supplementary Information:**

The online version contains supplementary material available at 10.1186/s13049-021-00932-z.

## Background

Mass casualty incidents (MCI), generate more casualties at one time than the locally available resources can manage using the routine procedures, highlighting that resource scarcity becomes a critical decision-making issue. MCI triage aims to prioritize casualties according to the severity of their injuries, often keeping available resources in mind [1–3]. Since WWI, utilitarianism has become a part of the triage systems in MCI. Basic utilitarianism states that an action is morally right if it produces maximal happiness or well-being for everyone affected by [4, 5]. Therefore, predetermined modern triage systems may unknowingly allow for the loss of a severely injured casualty’s life to provide treatment or evacuation to a greater number of casualties with lesser injuries [6]. This concept is equally used in hospital triage to justify which patients should receive treatment before others according to the alleged benefit of treatment [5–7]. Since 1964, when the first record of emergency department (ED) triage was described [8], the concept of triage has evolved into an even broader terminology, such as primary, secondary and tertiary triage, which can be subcategorized in diverse ways depending on a dynamic process where the patient’s vital signs can deteriorate over time [1, 9]. In general, triage categories can be expressed as a Description (immediate; Urgent; Delayed; Expectant), Priority (1 to 4), or Color (Red, Yellow, Green, Blue), respectively, where Immediate category equals Priority 1 and Red color [1, 2].

The distinction between the ordinary sorting and prioritizing of patients at a hospital ED from the more utilitarian prehospital triage of casualties from a natural disaster scene lies on the differences in available resources in the field versus that of a fully staffed hospital and may explain the notable variance seen between different, modern triage systems [1, 2, 5, 6, 9–11]. However, both hospital and prehospital triage systems may rely on four essential factors: speed, precision, fairness and compatibility [10]. For prehospital triage systems (especially in MCI), the element of speed of decision-making is of importance, since there are more casualties than the rescue staff can manage, and the post-incident environment might not be secure. Both hospital and prehospital triage also benefit from a fast, simple system that quickly identifies the patients or casualties that require an immediate intervention/evacuation [2, 5]. The element of speed cannot come at a too big of a sacrifice of precision, though. Generally, the more hastily the triage, the bigger the risk of faulty categorization. Under-triage increases the mortality among casualties because of a prolonged time to establish the accurate diagnosis and to provide proper interventions. Over-triage could lead to unnecessary consumption of resources, leading to increased morbidity and mortality [2, 11–17].

Prehospital systems allow for a more limited precision since speed remains frequently a priority while hospital triage can often sacrifice time to maximize precision. Fairness in triage is a matter of assessing patients objectively according to a set of parameters of vital signs or mechanisms of injury, and not discriminating in terms of age, gender, nationality, religion or any other individual aspect [1, 2]. Compatibility is applicable to triage systems being translational across agencies and to prehospital systems being able to integrate seamlessly with its hospital counterparts in terms of categorization, etc. [1, 2, 10, 15, 16]. Since its entry on the civilian stage in the 1960s, the area of triage has generated a plethora of diverse systems designed for equally as many situations, locations, nations and even within separate emergency medical services (EMS). These systems range from fast, crude algorithms and flowcharts to complex scoring systems requiring exact information on vital parameters, mechanisms of injury and available resources [6, 9, 11, 13, 17]. This heterogeneity constitutes a particular threat in the event of an MCI, which often involves rescue personnel from different organizations, or across-the-globe allies, and may create unnecessary discussion between EMS-personnel on the scene, haltering the time-critical management, timely triage decisions and evacuation of the victims [2, 18, 19]. The problem of variability has proven to be contentious and unsolvable often both immediately and for the long-term. There have been several attempts to achieve a global or even national consensus in a number of cases without fruition due to a lack of actual research behind the origin or refinements of the various systems. When proposing a modern system for universal consideration there has often not been much more than anecdotal evidence to its efficacy, making it hard to choose one over the other [15, 17, 20–23].

Diverse triage exist which leads to confusion. Therefore, a translational tool that combines criteria from different system is needed. This paper aims to take the first step in a multistep procedure to evaluate the possibility of creating a translational triage tool. The proposed tool, using criteria used in most common pre-existing prehospital triage systems, represents an applicable framework for a proposed unified global system. This approach acknowledges that existing systems are similar in terms of speed, precision and fairness and thereby tackles the compatibility element of triage. The majority criteria will reflect the intrinsic uniformity of present systems, highlighting the point in time whereby a global, translational system is accepted as the appropriate combination solution at the time.

## Methods

This rapid evidence review is the product of a qualitative meta-analysis of a number of identified triage systems [24]. The method consists of (1) a systematic literature review [25], (2) merging and content analysis of the studies focusing on similarities and differences between systems using the Constant Targeted Comparison process [26], and (3) the development of a universal operational system format/model. To avoid selection and opinion bias, each step of the methodology and decision-making was discussed, according to Nominal Group Technique (NGT) [27], in a group of three people (JN, EC, AK). AK and EC had extensive scientific and field experience (over 20 years) in both prehospital and hospital triage. JN was newly graduated physician, with no previous experience in triage systems.

Initially, a Preferred Reporting Items for Systematic Reviews and Meta-Analysis (PRISMA)-style literature search was conducted to identify the most frequently mentioned prehospital triage systems designed for use in MCIs [25]. To find an adequate, Boolean search term, a number of different keywords and combinations thereof were sampled with PubMed as a testbed. The search term “*((mass casualty incident) OR (multiple casualty incident)) AND (triage) AND ("emergency medical services")*” was selected. Using PubMed, Scopus and Web of Science databases for the final search, limiting and filtering of the search results was achieved through discussions among NGT members [27]. The search results were limited to articles and reviews published from 2000 to 2020, written in English. Further, in Web of Science and Scopus, the search was conducted with the option of searching ‘All Databases’ and additional filtering for just articles and reviews (under ‘Document Types’) was added. Identified duplicates were removed, following the PRISMA workflow-chart [25], by exporting all search results into Microsoft Excel, and using basic formatting to mark duplicate values and/or titles. The remaining articles’ title and abstracts were examined to identify suitable candidates for the final inclusion.

### Inclusions criteria

Articles focused on prehospital, primary triage in MCIs and on a triage system itself or a comparison of a number of systems.

### Exclusions criteria

Articles mentioning a triage system without any evaluation, or articles with different from the inclusion criteria. All articles about pediatric triage systems (since parameters found in these systems would be extreme outliers compared to the adult ones in the final mapping of overlapping criteria).

At this stage, all NGT group members reviewed, achieved consensus and approved the final selection of articles. In the final assembly of articles, abstracts were re-read and full texts studied, when needed, to determine which triage systems were mentioned. This resulted in a chart displaying the frequency of occurrence. From this chart, the top nine systems, mentioned more than once, were retrieved. The following step of the workflow chart from PRISMA constitutes further exclusions after reading each article in full text, each exclusion with its own written motivation and can be viewed as moving from the initial systematic search modus operandi towards a qualitative content analysis [26, 28, 29].

When a final assembly of systems had been decided on, the official sources or original articles of these systems were studied to collect data regarding the actual triage process of each system, often displayed in an accessible flowchart or algorithm. Of nine identified systems, two were discarded, since they were a part of a much larger system, which relied on further triages or a complex system where the categorization would be different from incident to incident depending on the available resources, and thus did not follow the traditional triage system layout. As such, an additional meta-synthesis was performed where flowcharts were translated into raw text, stating what exact criteria was demanded for a casualty to be placed within a certain category [26, 28–30]. The criteria of the remaining seven systems were then rephrased to further conform them into a translational dataset so that overlapping data could be displayed in a clear manner. The outcome could easily be separated into general subdivisions under each of the triage tiers. Two of the systems had five triage tiers instead of four like the others. These extra tiers were not added to the final compilation of parameters since they were obvious outliers. Under each subdivision, the individual parameters were collected and counted for in means of 1/7’s. When a final, compiled list of parameters with an equal phrasing had been assembled and counted for, a translated system was produced by combining the parameters of majority (≥ 4/7) under each triage tier.

## Results

**Fig. 1:**
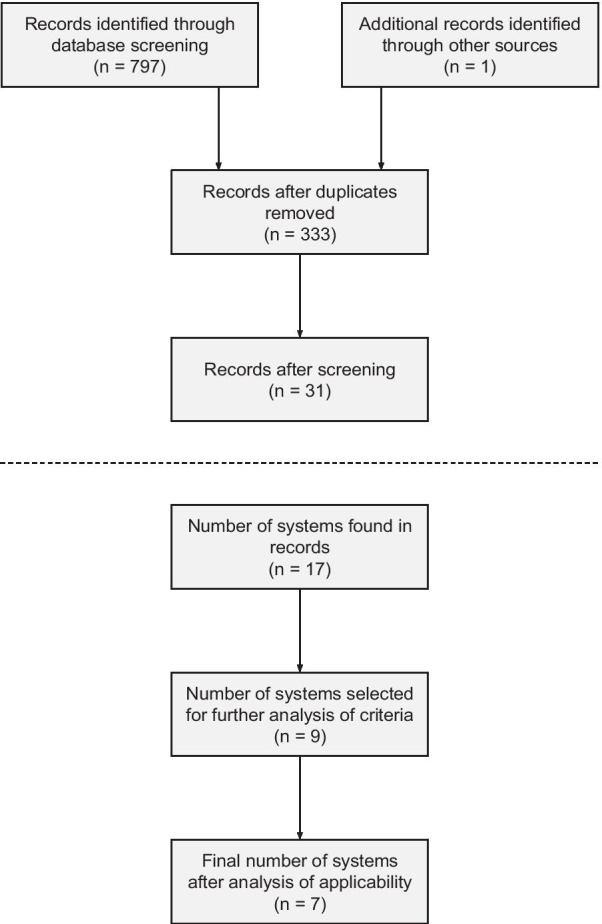
PRISMA-style workflow, depicting determination of both search and frequency of systems. The original PRISMA Flow Diagram (template) and the final list of records are accessible in Additional file 2: Appendix 2, and Additional file 3: Appendix 3, respectively

Additional file 1: Appendix 1 presents the primary testing of keywords, terms and combinations thereof. The search generated 797 hits (Web of Science: 287, PubMed: 266, Scopus: 244) with filters applied. The number of discarded duplicates, screened and included articles are demonstrated in Fig. 1 [14, 20, 31–60]. From the final list of 31 articles, 51 notification of 17 triage systems were found (Additional file 3: Appendix 3).

*1. Simple Triage And Rapid Transport (START), 2. Modified START (mSTART), 3. Fire Department of New York modified START (FDNY-START), 4. Modified Physiological Triage Tool (MPTT), 5. Amberg-Schwandorf Algorithm for Primary Triage (ASAV), 6. Sort Assess Lifesaving Intervention Triage/Transport (SALT), 7. CareFlight Triage (CFT), 8. Triage Sieve (TS), 9. Sacco Triage Method (STM), 10. Spanish Prehospital Advanced Triage Method (META), 11. Primary Ranking for Initial Orientation in Emergency Medical Services (PRIOR), 12. Field Triage Score (FTS), 13. Modified Military Sieve (mMS), 14. Military Sieve (MS), 15. Advanced Trauma Life Support (ATLS), 16. Glasgow Coma Scale (GCS), and 17. Chemical Biological Radiation Nuclear (CBRN).*

START and mSTART were counted as one since the difference between them only lies in the addition of controlling the radial pulse instead of performing the Blanch test (capillary refill) in cold conditions. Additionally, the two names are used interchangeably in the literature, often just calling the system START when it is the modified version that is referred to. Similarly, TS has undergone evolution to substitute capillary refill for heart rate in cold conditions, this addition did not affect the naming of the system and TS is used whether or not the modified version is the one being referred to. In contrast, MS and mMS were to be counted as two separate systems since they differ in criteria range in two out of three total assessments. This reasoning was of importance since it meant that MS and/or mMS were not selected for the final group of systems because they were mentioned once each, had they been counted for as one they would have made the cut.

The remaining top nine of the 17 systems were selected (START/mSTART, SALT, CFT, TS, STM, MPTT, FDNY, ASAV and META) for further analysis of actual system construction regarding criteria and categorization. As the analysis continued, two of the selected nine (STM and META) were identified as being non-compatible with the study design. STM is a mathematical model where individual vital parameters can yield different triage tiers from one event to another due to the available resources at that time, and META is a system where primary triage is closely integrated into a larger triage process. Picking the first step out of that concept and not acknowledging the rest of it seemed inappropriate. Additionally, the way both STM and META conducts its primary assessment of casualties differs considerably from the rest of the group. Consequently, seven systems remained for further analysis (Additional file 4: Appendix 4 a–g). The criteria from the final seven systems were collected in a spreadsheet and categorized according to system and inherent triage tier. Two of the seven systems had five triage tiers compared to the rest, which had four. The FDNY-START algorithm adds an ORANGE tier to easier identify GREEN and YELLOW casualties with the potential to medically deteriorate. According to the article of origin, ORANGE casualties also have an overrepresentation of chronic medical conditions, which could make decisions regarding transport easier [47]. The SALT system adds an EXPECTANT/GRAY tier. This tier consists of IMMEDIATE/RED casualties who are not likely to survive given current resources [21].

The criteria from each system were then rephrased to enable comparability and overlapping [26]. As all criteria had been through a first rephrasing, obvious merging was identified and performed (Additional file 5: Appendix 5) [26, 28, 29]. For example, the criterion “*Not breathing with an open airway*” was merged with “*Airway not open*”, “*Decapitated, dismembered, transection of torso*” with “*Decapitation or destruction of the torso*” and “*Radial pulse absent*” with “*Peripheral pulse absent*” and so forth. The largest merge was done in the YELLOW/URGENT/PRIORITY 2 tier. Since all systems were chronological flowcharts with initial division due to ambulation and with all criteria laid out it was recognized that YELLOW casualties simply could be summarized by the criterion “*Non-ambulatory (and not [black (PX)] or [red (P1)])*”. This was done instead of displaying every opposite criteria of the RED tier under YELLOW. For example, “Radial/peripheral pulse *absent* = RED, Radial/peripheral pulse *palpable* = YELLOW” etc. just poses as information overflow. Instead, all YELLOW criteria (except one) could be compiled under the merged phrase above. With all criteria rephrased so that they could be quantified, the criteria were collected and counted for in means of 1/7’s (Table 1). The extra tiers from FDNY-START and SALT did not undergo rephrasing since these were obvious outliers. The ORANGE tier of FDNY-START was also excluded based on that; it is a form of *secondary* assessment, meant to be used after casualties has been tagged as either YELLOW or GREEN. Looking at the majority criteria of each triage tier, using the casualty’s ability to ambulate or not as a primary regulator to sort out DELAYED/PRIORITY 3 (P3) casualties from the rest was apparent as it was represented in 6/7 systems. The only system that did not contribute to the ambulatory criterion, SALT, actually uses it but in a step that foregoes the individual triage of casualties.

**Table 1:** Rephrased and merged criteria with fractions

***DEA/PX***	**Non-ambulatory (5/7)** **Not breathing (7/7)** after 1–2 attempts at positioning airway (3/7) after chest decompression (1/7) No pulse (1/7) Obvious signs of death (2/7) Following commands/neurological status (1/7) unable (1/7)
*IMMEDIATE/P1*	**Non-ambulatory (6/7)** **Breathing/Open airway (6/7)** only after positioning (1/7) Respiratory distress (2/7) **RR (4/7)** > 30/min (1/7) ** > *30/min or* < *10/min (1/7*) (full coverage)** ≥ 22/min or < 12/min (1/7) > 29/min or < 10/min (1/7) **Radial/peripheral pulse absent (5/7)** HR (2/7) ≥ 100/min (1/7) > 120/min (1/7) CF > 2 s (2/7) **Following commands/neurological status (6/7)** **unable (5/7)** …or not making purposeful movements (1/7) GCS < 14 (1/7) Major hemorrhage persistent after attempt to control (2/7) Likely to survive given current resources (1/7) Not deadly injured (1/7)
*URGENT/P2*	**Non-ambulatory and not [PX] or [P1] (6/7)** Not fulfilling any [PX] or [P1] criteria (1/7) More than minor injuries (1/7)
*DELAYED/P3*	**Ambulatory (6/7)** Only minor injuries (1/7)

Majority criteria shown in bold letters

This first step is called “Global sorting” and states that casualties that are able to walk should be assessed third after those able to wave/do purposeful movements (2^nd^) and still/casualties with an obvious life threat (1^st^) [21]. Rephrasing URGENT/PRIORITY 2 [P2] criteria to “*Non-ambulatory (and not [PX] or [P1])*” was successful in 6/7 systems. SALT being the marginal outlier again, since P2 criteria could be rephrased as “*Not fulfilling any [PX] or [P1] criteria (1/7)*”, ambulation being left out because of the same reason as above.

A wider spectrum of criteria could be found in the IMMEDIATE/PRIORITY 1 [P1] tier. Rephrasing managed to isolate five main criteria: “Non-ambulatory” (6/7), “Breathing/Open airway” (6/7), “Respiratory rate (RR)” (4/7), “Radial/Peripheral pulse absent” (4/7) and “Following commands/neurological status” (6/7). Counting a respiratory rate without knowing the determining interval is pointless which is why the four suggested intervals were studied closer. Three out of 4 intervals suggested both a lower and a higher limit. Having noted this, the one interval that covered the whole spectrum of suggested limiters was selected as a criterion for P1: RR < 10 or > 30/min. The criterion “Following commands/neurological status” had a subordinated specification which was in majority: “Unable” (4/7), differentiating it from other ways to assess neurological status.

**Fig. 2:**
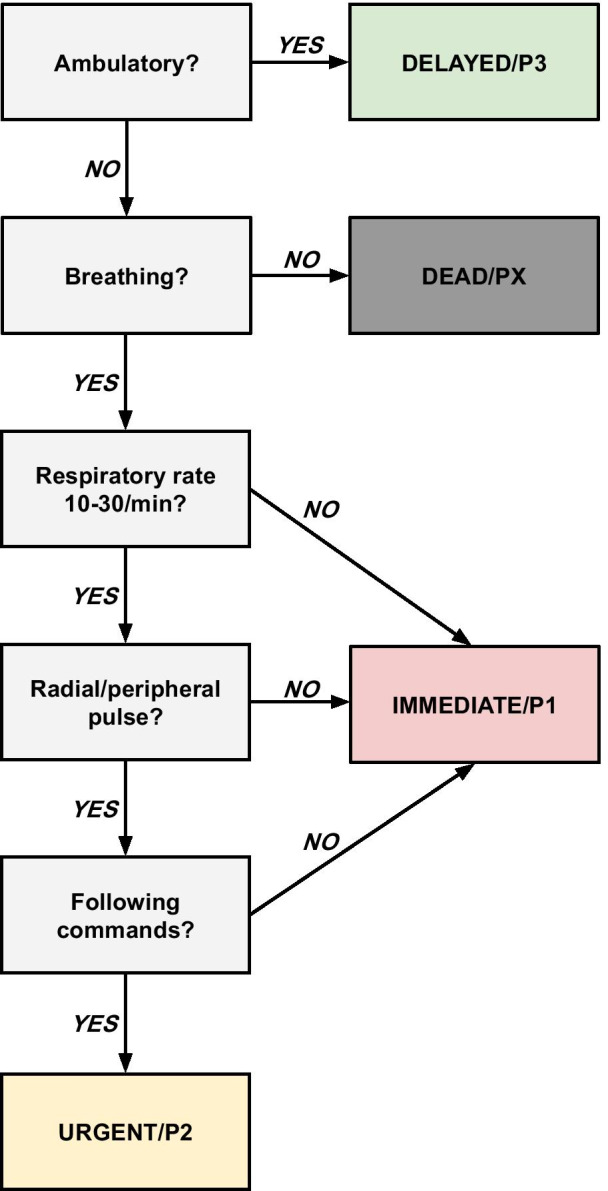
System constructed from majority criteria

In the DEAD/PRIORITY X [PX] tier, two determining criteria were found: “Non-ambulatory” (6/7) and “Not breathing” (7/7). Assessing the ability to walk to determine whether a casualty is to be categorized as dead might appear inappropriate but since ambulation was the majority criterion to single out P3 casualties, all subsequent tiers would have the negated version of that criterion. All systems used breathing or assessment of the airway [with or without intervention] as a determiner for PX. As a final step, a mock system was constructed of the criteria that were in majority (≥ 4/7) as a way to illustrate the findings (Fig. 2). The derivation of each step of the final system can be found in the Table 2.

**Table 2:** Majority criteria and the system they were derived from

*CRITERIA*	*DERIVED FROM SYSTEM*
Ambulatory: *YES/NO?*	START/mSTART, FNDY-START, MPTT, ASAV, CFT, TS
Breathing: *YES/NO?*	START/mSTART, FNDY-START, MPTT, ASAV, SALT, CFT, TS
Respiratory rate	START/mSTART, FDNY-START, MPTT, TS
10-30/min: *YES/NO?*	FNDY-START
Radial/peripheral pulse: *YES/NO?*	START/mSTART, FNDY-START, ASAV, SALT, CFT
Following commands: *YES/NO?*	START/mSTART, FDNY-START, ASAV, SALT, CFT

## Discussion

This paper aims to take the first step in a multistep procedure to evaluate the possibility of creating a translational triage tool for prehospital use in mass casualty incidents. Doing so, it also creates a discussion regarding the current use of prehospital triage and number-based prehospital decision-making. The power of triage goes, however, behind the numbers and lies on the ability of providers to recognize and decode clinical symptoms [1]. Having this in mind, the continuous emergence of new triage systems may witness the lack of a reliable solution and flaws in all prehospital protocols, which neither may be translated into true clinical value and diagnostic substance, nor demonstrate evidence or validity [20, 61]. Nevertheless, they definitely demonstrate organizational and management advantages and should be adjusted, if possible [62].

The combined criteria system, discussed in this paper, should be considered as a way to display the results and not as an actual proposal for yet another triage system. Below, a breakdown of each segment of the combined system, which leads to a translated version, is described. This version, discussed in reference to relevant research, is the true product of the methodology suggested in this study.

### Ambulation

As the primary divider, sorting out the least prioritized casualties from the rest is a very common criterion in prehospital triage systems aimed at handling greater numbers of casualties [17]. A casualty commanded to walk to a secure rendezvous point, must have sufficient central nervous system (CNS) function to receive, process and elicit a motor response to the command. This also implies that there is sufficient blood pressure to sustain the move. Thus, the victim cannot be suffering from severe structural damages making movement impossible. This concept is incorporated in so many systems. It is important to remember that this study is limited to *primary* triage. If possible, a second assessment must be made as soon as casualties arrive at a designated collecting area since initially ambulatory casualties stand a risk of deteriorating quickly [6, 10, 11, 13].

### Breathing/open airway

As a deciding factor for categorizing casualties as PX is also a common criterion in MCI triage [17]. The phrasing differs, but the general idea is that not breathing by itself means that the resources required for its resuscitation cannot be guaranteed in the event of an MCI, irrespective of the causes. Several systems allow for 1–2 attempts of a lifesaving intervention [LSI] at this stage [1, 6]. The hierarchy of assessing the airway first is a well-proven concept, stemming from the training program Advanced Trauma Life Support (ATLS) [63]. In simple terms, if the brain cannot get oxygen, the other organ systems will not function leading to death.

### ***Respiratory rate*** (RR)

Coincides with the second point of assessment of the ATLS system (B—Breathing and ventilation). RR is instrumental for finding casualties with primary injuries affecting the airways/lungs, such as direct trauma to the thorax (hemo-/pneumothorax) or inhalation burns/smoke related injuries that are breathing and can be saved. They may also have secondary injuries, such as major hemorrhage (hypovolemia) resulting in a physiological response manifesting as tachypnea. These patients manifest with a deviant breathing pattern [64]. Therefore, it makes sense that injuries with altered breathing patterns have a higher priority to receive immediate attention [63, 64]. Nevertheless, assessing the breathing by counting respirations per minute has obvious shortcomings.

Firstly, the counting cannot be done rapidly since it is not entirely binary in its result (as in: “*Breathing: YES/NO?*”) [10]. It may also take several minutes to count respirations in a conceivably loud, stressful and weather affected surrounding [13]. Furthermore, the RR might be very dynamic in its frequency so that the rate, when being assessed, does not give a fair picture of the actual condition of the casualty. The casualty being assessed may also be under severe psychological stress from the MCI itself resulting in tachypnea or hyperventilation that does not come from life threatening injuries, or could be in psychological shock from realizing that it is severely injured, which, on its own or in combination with the injury, drives a psychogenic hyperventilation [64–67]. Counting an RR also demands an interval of acceptable values. No matter what the chosen interval is, there is always gray areas (compare the casualty with RR = 29/min vs > 30/min). The interval chosen for the final system was just the interval that covered the whole spectrum of the four alternatives (*START/mSTART: no lower limit, just* > *30/min, FDNY-START; 10–30/min, MPTT: 11–22/min, and TS: 9–30/min*) [13, 45, 46, 64].

Although the value of RR is to diagnose whether the victim is brady- or tachypneic, it would be more valuable to know if the breathing is compromised in ways other than frequency (dyspneic or sudden apneic episodes, wheezing, severe coughing with expectoration etc.). This could all be summarized in a criterion with a binary answer: “*Respiratory distress: YES/NO?*” which would be more aimed at assessing the quality of breathing instead of the *quantity*. This idea can be recognized in two of the systems in the final group, SALT and ASAV. SALT poses the question “*Not in respiratory distress?*” while ASAV asks “*Breathing difficulties?*” with a short definition written in close proximity to the main flowchart [21, 49].

In conclusion, taking the time to assess an RR with a somewhat arbitrary interval of acceptable values merely results in an assessment of quantity of breathing at that precise moment. Quickly assessing if the casualty is in respiratory distress or not seems favorable considering both time consumption and sensitivity to detect life-threatening injuries. It does, on the other hand, require some level of basic medical training to judge if a person is in respiratory distress or not, leaving out the possibility for other personnel (than medically trained) to conduct the triage.

### Radial/peripheral pulse

Is a proxy used to estimate blood pressure and coincides well with the third level of assessments in the ATLS system (C = Circulation with bleeding control). An estimation of blood pressure is of value since a hypotensive trauma casualty has a high risk of life-threatening external or internal hemorrhage. The matter of hypotension from hypovolemia gives a twofold yield in trauma casualties:

1. Casualties with life-threatening internal bleeding that needs to be tagged as P1 for immediate evacuation and surgical intervention at a hospital.
2. Casualties with life-threatening external bleeding that hardly needs their radial pulse checked to be assessed (gushing arterial bleeding tends to be obvious). They need an intervention in the field (see LSI’s below), immediate evacuation and surgical intervention at a hospital.

Historically, a palpable radial pulse has been equated to a systolic blood pressure (SBP) of > 80–90 mmHg [1, 13, 18, 45] and an SBP of < 90 mmHg is widely taught as a sign of shock [68] or the limit of clinical hypotension. The broad term of shock is applicable to the trauma casualty primarily as a function of hypovolemia due to major hemorrhage. Hemorrhage-induced hypotension in trauma patients has been found to be a predictor of significant mortality [69]. The main reason to assess the radial pulse is to ask the question: “*Hypotension (due to hypovolemia): YES/NO?*” The idea of a palpable radial pulse being equitable to an SBP of > 80 mmHg stems from the first edition of the ATLS program, published in 1985 [70]. The program states that a palpable carotid pulse equals an SBP of 60–70 mmHg, if a femoral pulse can be palpated as well then the SBP lies somewhere between 70–80 mmHg and a palpable radial pulse equals an SBP of > 80 mmHg. This paradigm was quickly rejected [71] due to the lack of data to support it and the guidelines were removed from subsequent iterations of the program. Somehow, though, these ideas of pulse palpability and its proxy as levels of SBP has stuck throughout the decades since and is still today a subject of discussion and controversy [64, 72–75]. Another issue lies in the fact that some sources suggest that an affected SBP is a very late sign of hypovolemia, implying that when changes occur, it might be too late for successful interventions, especially in the prehospital setting [76]. The alternative, to time capillary refill in seconds (as in the earlier iterations of START and TS) is very condition dependent (cold weather, poor lighting) and has also been considered a highly insufficient substitute for blood pressure for decades [77].

Uncontrolled hemorrhage translates into a significant amount of civilian trauma-associated and military battlefield deaths. Numbers as high as 80% of civilian trauma deaths has been linked to uncontrolled hemorrhage while the same source account up to 50% of deaths on the battlefield to the same [78]. Another source looked at deaths on the battlefield from 2001 to 2011 and found that 24.3% were potentially survivable injuries whereby 90.9% were associated with hemorrhage [79]. All of this obviously supports *some* form of blood pressure assessment in the MCI triage. To demand the use of a sphygmomanometer in the event of an MCI is out of the question. Not only does it require a stethoscope (and quiet surroundings) in addition to the blood pressure cuff, it also takes an impractical amount of time [78]. Since no other assessment of blood pressure is available without equipment, the palpation of the radial pulse will probably keep its place in future prehospital triage systems. At least, it seems rational to prioritize a casualty *without* a palpable radial pulse higher than one with a palpable one. To differentiate the casualties with a high probability of an internal bleed from the ones that need an intervention in the field (life-threatening external bleed), an additional question could be added, as described in the ASAV and SALT systems [21, 49]. First asking for: “*Major external bleeding: YES/NO?*” and if: YES, proceed with an LSI while if: NO, proceed with “*Radial pulse palpable: YES/NO?*”.

### Following commands

Also follows the ATLS algorithm, matching the fourth step (D—Disability/Neurologic assessment). The seemingly elementary assessment of whether a casualty can follow commands or not actually tells us a number of things. Someone who can follow a simple command, such as, “Can you show me where it hurts?”, “Can you tell me where you are?” or merely “Can you wave your hand at me?” can receive and process auditory information and then turn it into a verbal or motor response. Consequently, in this patient neither is the flow of oxygen critically impaired, nor is there substantial structural damage to the CNS or the routes thereto. This assessment of verbal or motor response to stimuli is a part of the Glasgow Coma Scale (GCS) [79], which evaluates the level of consciousness in patients with acute brain injury. Especially the motor component of the scale has been found to be a predictor of both the need for an LSI (together with a weak or absent radial pulse) [77] or the risk of dying [80, 81] in studied groups of trauma patients. The criterion to follow simple commands was supported in some way or another in all the final seven systems and can be found in several other triage systems [17] that were not included in this analysis.

### Lifesaving interventions

Were included in 4/7 systems in the final group (Table 3). The question of LSIs or not in a triage system for MCI’s translates to two sub-questions: (1) who will perform the triage, and (2) what resources/equipment are available in the field?

**Table 3:** Systems and LSIs

*SYSTEM*	*LSI*
START/mSTART	Positioning and repositioning of airway (if needed)
FDNY-START	Opening of airway
MPTT	None
ASAV	Keeping airway open
	Stopping bleeding
SALT	Controlling major hemorrhage
	Opening airway (2 rescue breaths if child)
	Chest decompression
	Auto injector antidotes
CFT	None
TS	None

The answer to the first question is somewhat a matter of triage philosophy. Should triage be strictly limited to medical personnel or should volunteers or even bystanders be able to help [82]? Several countries have legislation concerning who exactly can make such decisions regarding life and death. In addition, from an ethical point of view, should someone without medical training and insight into available resources be responsible for making such choices? Dealing with the pressure of decision-making lies in the simplicity of the triage system. A triage system in its simplest form should be usable for anyone but that would require a redundancy level that might not provide correct prioritization from a strictly medical point of view. Correspondingly, a system that is so uncomplicated that *anyone* could conduct the triage leaves absolutely no room for LSIs to be performed in the field, although not all LSIs are equal, concerning what medical training they require. Looking at the LSIs in Table 3, using an antidote auto-injector is an elementary intervention as long as it comes with an accessible manual of operation and instructions of acceptable injection sites. However, the decision that all casualties should receive an antidote must come from a medical professional. Controlling major hemorrhage by applying a tourniquet [82, 83] or direct pressure to an extremity is also seemingly straightforward, while attempting to stop a gushing abdominal wound in a satisfactory manner requires surgical knowledge. The same applies for airway opening and control, to position the casualty’s head adequately, is not necessarily complicated, but to judge whether the positioning is good enough to tag a casualty as PX when breathing does not start is a decision to make for someone with extensive medical training. Performing a needle or mini thoracostomy in the field to decompress a suspected hemo- or pneumothorax ranks as the most complex of the LSIs in the table above. This demands highly skilled professional to perform, definitive medical training, sufficient management conditions and equipment.

The answer to the second question is closely linked to the first. As stated earlier, a triage system for MCIs must be constructed with austere conditions and minimal equipment requirements in mind. This is reflected in the table of LSIs; the positioning of the airway is the LSI in majority since it is performed without equipment to prevent an obstructed airway and is a rapid intervention. As described above, major hemorrhage accounts for a substantial amount of preventable trauma deaths, which warrants at least an attempt at controlling the bleeding on prehospital scene. This could also be made with minimal equipment; makeshift tourniquets can be made from clothing, direct pressure can be applied by bystanders, another victim or, in some cases, even the injured casualty [82].

**Fig. 3:**
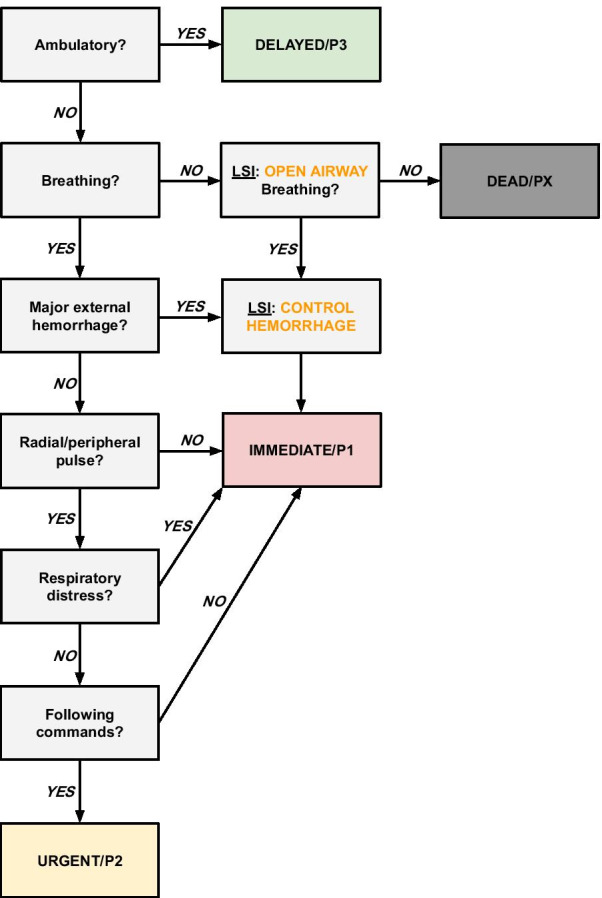
Triage System constructed from majority criteria. Modified according to discussion regarding triage criteria and LSIs

Field packing of wounds to the abdomen or proximal portion of limbs ranks higher not only in surgical training required but also in equipment demand and time consumption. The matter of antidote administration depends on regional resources, stockpiles and the fact that the contaminating agent has to be identified. Performing needle or mini thoracostomy as an LSI in the triage algorithm would require professional training and necessary equipment. The most LSI-heavy system of the final seven, SALT, states that no LSI should be performed without sufficient medical training and resources [21]. Adding this disclaimer to a future combined triage system is a way around the restrictions of LSIs. Since MCI triage is traditionally aimed at medical personnel conducting it, at least airway positioning and controlling of hemorrhage should be allowed. To cover contaminated events further, a future system should also allow for rapid administration of antidotes when needed. Concerning a system constructed out of majority criteria: a more nuanced system would probably emerge from research with greater resources that could include a wider array of existing triage systems. The value of the different stages of the algorithm can be justified at the moment of decision-making. The translated triage version in this paper (Fig. 3) should be viewed as a final step in the methodology of this paper.

### Chemical, biological, radiation and nuclear (CBRN)

**Fig. 4:**
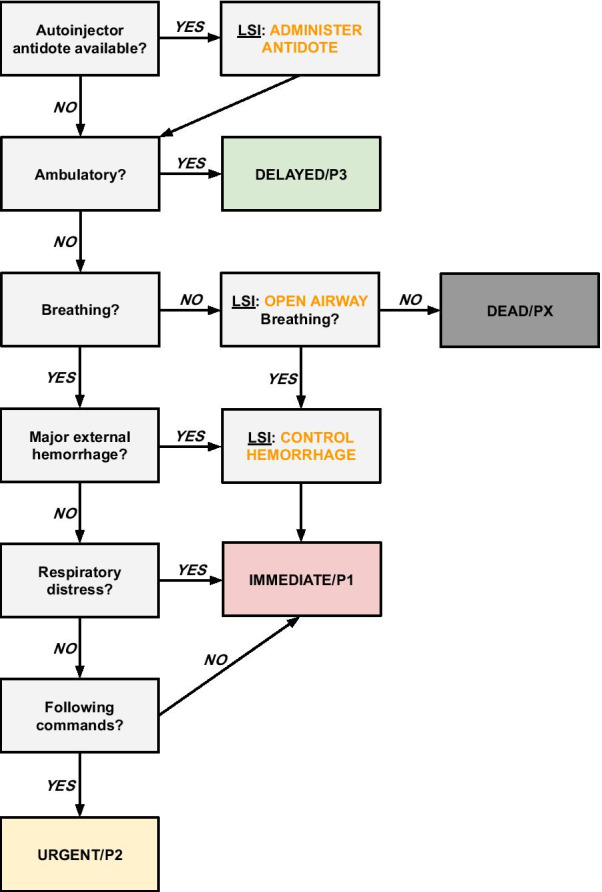
Triage System constructed from majority criteria, modified according to discussion regarding criteria, LSIs, and CBRN variation

CBRN is a frequently used term when discussing triage in the MCI setting. Adding a layer of contamination of both casualties and incident site to the triage process complicates it exponentially. Several currently widespread MCI triage systems have not been studied for use in these situations [20, 48, 84]. A number of specific triage systems for CBRN exist [17] but none of these made the final selection in this analysis. Of the final seven, only SALT lists a specific intervention that could be linked to a CBRN-scenario; the administration of antidotes. However, all MCI triage systems analyzed here depend on vital parameters or physiological criteria for the prioritization of casualties. It seems plausible that the CBRN casualty would have equally as deranged vital parameters as the trauma counterpart. Instead, the CBRN-scenario’s prime challenging factor concerning primary triage is that it would have to be conducted wearing hazardous material (hazmat) protection gear, making some assessments impossible. Using the proposed translated triage, the problematic part will still be counting a respiratory rate, certainly not when aerosol or gas-form irritant or blistering agents have been used, because of coughing, wheezing, psychogenic tachypnea etc. Again, identifying *respiratory distress* to assess *quality* of breathing instead of *quantity* is the appropriate route to go. Palpating for a radial pulse would be hard if not possible at all. Applying a tourniquet or some other ad-hoc solution to stop major external hemorrhage, is probably possible, keeping the suggested question of “*Major external hemorrhage: YES/NO?*” in the CBRN application of the modified combined criteria system. Verbal communication might be impaired when using respiratory protection apparatuses but to gain a general idea of whether the casualty is able to follow commands seems imaginable (Fig. 4). Envisioning the application of any MCI triage system in a CBRN-setting is easy in theory but requires extensive, further research and input from both civilian and military experts.

### Limitations

The main limitation of this study is in the number of studies reviewed. It is also evident that reviewing only English literature, may result in missing studies from other countries. The PRISMA-workflow was not followed precisely since the aim of the study was not a systematic review. Consequently, no attention was given to the quality of the articles, impact factor etc. However, a large portion of the methodology was chosen to identify and justify *any* scientifically proven way to select and warrant the selection of the systems instead of just picking out a specific number. Another limitation goes to the small group of systems selected for the final analysis of overlapping criteria. A majority of the final seven systems stem from the original START system, making for a relatively homogenous group, which leads to a START-like preference product in the end. If a larger selection of articles could have been made from a broader original search then a larger, more diverse group of systems could have been compared. In this scenario, the overlapping and the final system might have looked different. In addition, restricting the rephrasing and overlapping to four tiers (as in excluding ORANGE and GRAY tiers from FDNY-START and SALT) could have contributed to a uniform end-result.

Future research could incorporate any number of tiers to see if there are overlapping criteria in the non-classical categories as well. Finally, constant targeted comparison was used as preferred analytic device to create meta-syntheses of findings. The method deliberately searches for similarities and differences between a target phenomenon and some other extra-study phenomenon, with obvious alikeness [26]. Such comparison aims to clarify the defining and overlapping characteristics of the target phenomenon in order to minimize the likelihood of inflating its uniqueness and to help ascertain the relationships between phenomena and is best conducted after reducing the findings in all reports into a set of abstracted statements, or represented in taxonomy, as conducted in this study.

## Conclusion

The current study demonstrates two essential points. To begin with, there are common characteristics in all current prehospital triage systems, and, secondly, it is possible to substitute the number-based prehospital decision-making with clinical signs and symptoms. Accordingly, it seems to be feasible to convert various prehospital triage systems into one by using a triage translational tool. However, this study is the first step in a multistep future research that should examine the tool and its different decision-making steps either by using simulations or by experts’ evaluation to ensure its feasibility in terms of speed, continuity, simplicity, sensitivity and specificity. The used methodology may not follow the existing tradition, however, applying this model to further, broader research; including a wider array of more heterogeneous triage systems has the potential to result in a translational triage tool for worldwide use.

## Supplementary Information

**Additional file 1.Appendix 1:** Presents the primary testing of keywords, terms and combinations thereof.

**Additional file 2.Appendix 2:** PRISMA-style workflow, depicting determination of both search and frequency of systems.

**Additional file 3.Appendix 3:** The final list and frequency of the literature.

**Additional file 4.Appendix 4:** The Final Seven Systems included in this study (a–g).

**Additional file 5.Appendix 5:** Original phrasing, rephrasing and merging of criteria from the final seven systems.

## Data Availability

All data generated or analyzed during this study are included in this published article or as supplementary files. References used in the literature review are available on world web.

## Abbreviations

ASAV: Amberg–Schwandorf algorithm for primary triage
ATLS: Advanced trauma life support
CBRN: Chemical biological radiation nuclear
CFT: Care flight triage
CNC: Central nervous system
ED: Emergency department
EMS: Emergency medical services
FDNY-START: Fire Department of New York modified START
FTS: Field triage score
GCS: Glasgow Coma Scale
LSI: Life saving intervention
MCI: Mass casualty incidents
META: Spanish prehospital advanced triage method
mMS: Modified military sieve
MPTT: Modified physiological triage tool
MS: Military sieve
mSTART: Modified START
NGT: Nominal group technique
P: Priority
PRIOR: Primary ranking for initial orientation in emergency medical services
PRISMA: Preferred reporting items for systematic reviews and meta-analysis
RR: Respiratory rate
SALT: Sort assess lifesaving intervention triage/transport
Start: Simple triage and rapid transport
STM: Sacco triage method
TS: Triage sieve
WWI: World War I
