# Supplementary material for: A translational triage research development tool: standardizing prehospital triage decision-making systems in mass casualty incidents
Source: Scand J Trauma Resusc Emerg Med. 2021 Aug 17;29:119. doi: 10.1186/s13049-021-00932-z (PMC8369703; doi:10.1186/s13049-021-00932-z)
Supplement: Supplementary file 1 — Additional file 1.Appendix 1: Presents the primary testing of keywords, terms and combinations thereof. [file 13049_2021_932_MOESM1_ESM.docx]

**Appendix 1: Presents the primary testing of keywords, terms and combinations thereof.**
