# Supplementary material for: A translational triage research development tool: standardizing prehospital triage decision-making systems in mass casualty incidents
Source: Scand J Trauma Resusc Emerg Med. 2021 Aug 17;29:119. doi: 10.1186/s13049-021-00932-z (PMC8369703; doi:10.1186/s13049-021-00932-z)
Supplement: Supplementary file 2 — Additional file 2.Appendix 2: PRISMA-style workflow, depicting determination of both search and frequency of systems. [file 13049_2021_932_MOESM2_ESM.doc]

**Appendix 2: PRISMA-style workflow, depicting determination of both search and frequency of systems.**

**Screening**

**Included**

**Eligibility**

**Identification**

Records identified through database searching
(n = 797)

Additional records identified through other sources
(n = 1)

Records after duplicates removed
(n = 333)

Records screened
(n = 333)

Records excluded
(n = 302)

~~Full-text articles assessed for eligibility
(n = )~~

~~Full-text articles excluded, with reasons
(n = )~~

~~Studies included in qualitative synthesis
(n = )~~

~~Studies included in quantitative synthesis (meta-analysis)
(n = )~~
