# Supplementary material for: A translational triage research development tool: standardizing prehospital triage decision-making systems in mass casualty incidents
Source: Scand J Trauma Resusc Emerg Med. 2021 Aug 17;29:119. doi: 10.1186/s13049-021-00932-z (PMC8369703; doi:10.1186/s13049-021-00932-z)
Supplement: Supplementary file 3 — Additional file 3.Appendix 3: The final list and frequency of the literature. [file 13049_2021_932_MOESM3_ESM.docx]

**Appendix 3: The final List and frequency of the literature.**
