# Supplementary material for: A translational triage research development tool: standardizing prehospital triage decision-making systems in mass casualty incidents
Source: Scand J Trauma Resusc Emerg Med. 2021 Aug 17;29:119. doi: 10.1186/s13049-021-00932-z (PMC8369703; doi:10.1186/s13049-021-00932-z)
Supplement: Supplementary file 4 — Additional file 4.Appendix 4: The Final Seven Systems included in this study (a–g). [file 13049_2021_932_MOESM4_ESM.docx]

**Appendix 4: The Final Seven Systems included in this study (a-g).**

**a. Simple Triage and Rapid Transport (START) and Modified START (mSTART)**

*Source: Community Emergency Response Team Unit, L. A. F. D. H. S. D. Simple Triage and Rapid Treatment (START), <https://www.cert-la.com/cert-training-education/start/> (2020).*

**b. Fire Department of New York modified START (FDNY-START)**

*Source: Arshad, F. H. et al. A modified simple triage and rapid treatment algorithm from the New York City (USA) Fire Department. Prehosp Disaster Med 30, 199-204, doi:10.1017/S1049023X14001447 (2015).*

**c. Modified Physiological Triage Tool (MPTT)**

*Source: Vassallo, J., Beavis, J., Smith, J. E. & Wallis, L. A. Major incident triage: Derivation and comparative analysis of the Modified Physiological Triage Tool (MPTT). Injury 48, 992-999, doi:10.1016/j.injury.2017.01.038 (2017).*

**d. Amberg-Schwandorf Algorithm for Primary Triage (ASAV)**

*Source: Wolf, P., Bigalke, M., Graf, B. M., Birkholz, T. & Dittmar, M. S. Evaluation of a novel algorithm for primary mass casualty triage by paramedics in a physician manned EMS system: a dummy based trial. Scand J Trauma Resusc Emerg Med 22, 50, doi:10.1186/s13049-014-0050-6 (2014).*

**e. Sort, Assess, Lifesaving Intervention, Triage/Transport (SALT)**

*Source: Lerner, E. B. et al. Mass casualty triage: an evaluation of the science and refinement of a national guideline. Disaster Med Public Health Prep 5, 129-137, doi:10.1001/dmp.2011.39 (2011).*

**f. CareFlight Triage (CFT)**

*Source: Garner, A., Lee, A., Harrison, K. & Schultz, C. H. Comparative analysis of multiple-casualty incident triage algorithms. Ann Emerg Med 38, 541-548, doi:10.1067/mem.2001.119053 (2001).*

**g. Triage Sieve (TS)**

*Source: Garner, A., Lee, A., Harrison, K. & Schultz, C. H. Comparative analysis of multiple-casualty incident triage algorithms. Ann Emerg Med 38, 541-548, doi:10.1067/mem.2001.119053 (2001).*
