# Supplementary material for: A translational triage research development tool: standardizing prehospital triage decision-making systems in mass casualty incidents
Source: Scand J Trauma Resusc Emerg Med. 2021 Aug 17;29:119. doi: 10.1186/s13049-021-00932-z (PMC8369703; doi:10.1186/s13049-021-00932-z)
Supplement: Supplementary file 5 — Additional file 5.Appendix 5: Original phrasing, rephrasing and merging of criteria from the final seven systems. [file 13049_2021_932_MOESM5_ESM.pdf]

APPX5_PHRASE_REPHRASE_MERGE

System, Source	Tier	Original phrase	Rephrasing	Merging with/final form
**START/<br>mSTART**<br><br>*Community Emergency Response Team Unit, L. A. F. D. H. S. D. Simple Triage and Rapid Treatment (START), <https://www.cert-la.com/cert-training-education/start/> (2020).*	**DEAD**	Non-walking	Non-ambulatory	Non-ambulatory
		Respiration: NO<br>_After positioning and repositioning of airway: NO	Not breathing after 2 attempts of positioning airway	Not breathing<br>_after 1-2 attempts of positioning airway
	**IMMEDIATE**	Non-walking	Non-ambulatory	Non-ambulatory
		Respiration: NO<br>_After positioning of airway: YES/NO<br>_After respositioning of airway: YES	Breathing only after positioning of airway (1 or 2 tries)	Breathing/open airway<br>_only after positioning
		Respiration: YES<br>_Over 30/min	RR >30/min	RR<br>_>30/min
		Radial pulse: Absent (mSTART)	Radial pulse absent	Radial/peripheral pulse absent
		Blanch test: Over 2/sec (START)	CF >2 sec	CF >2 sec
		Mental status: Can't follow simple commands	Unable to follow commands	Following commands/neurological status<br>_unable
	**DELAYED**	Non-walking	Non-ambulatory	Non-ambulatory and not [black (PX)] or [red (P1)]
		Respiration: YES<br>_Under 30/min	Not fulfilling any [black] or [red] criteria	
		Radial pulse: Present (mSTART)		
		Blanch test: Under 2/sec (START)		
		Mental status: Follows simple commands		
	**MINOR**	Walking wounded and uninjured	Ambulatory	Ambulatory
**FDNY-START**<br><br>*Arshad, F. H. et al. A modified simple triage and rapid treatment algorithm from the New York City (USA) Fire Department. Prehosp Disaster Med 30, 199-204, doi:10.1017/S1049023X14001447 (2015).*	**BLACK**	Non-ambulatory	Non-ambulatory	Non-ambulatory
		Obvious signs of Death: Decapitated, Dismembered, Transection of Torso	Obvious signs of death (defined)	Obvious signs of death
		Breathing: No, even after opening airway	Not breathing after 1 attempt at positioning airway	Not breathing<br>_after 1-2 attempts of positioning airway
	**RED**	Non-ambulatory	Non-ambulatory	Non-ambulatory
		Breathing: YES	Breathing	Breathing/open airway
		Respirations rate 10 to 30: NO	RR>30/min or RR<10/min	RR<br>_>30/min or <10/min
		Radial pulse: NO	Radial pulse absent	Radial/peripheral pulse absent
		Follows commands: NO	Unable to follow commands	Following commands/neurological status<br>_unable
	**YELLOW**	Non-ambulatory	Non-ambulatory	Non-ambulatory and not [black (PX)] or [red (P1)]
		Respirations rate 10 to 30: YES	Not fulfilling any [black] or [red] criteria	
		Radial pulse: YES		
		Follows commands: YES		
	**GREEN**	Ambulatory	Ambulatory	Ambulatory
	**ORANGE**	GREEN or YELLOW casualty who subsequently is found to have:<br>- Respiratory Distress<br>- Labored Respirations<br>- Change in Mental Status<br>- Head Trauma<br>- Chest Pain<br>- Index of Suspicion?	GREEN or YELLOW casualty who subsequently is found to have:<br>- Respiratory Distress<br>- Labored Respirations<br>- Change in Mental Status<br>- Head Trauma<br>- Chest Pain<br>- Index of Suspicion?	GREEN or YELLOW casualty who subsequently is found to have:<br>- Respiratory Distress<br>- Labored Respirations<br>- Change in Mental Status<br>- Head Trauma<br>- Chest Pain<br>- Index of Suspicion?
**MPTT**<br><br>*Vassallo, J., Beavis, J., Smith, J. E. & Wallis, L. A. Major incident triage: Derivation and comparative analysis of the Modified Physiological Triage Tool (MPTT). Injury 48, 992-999, doi:10.1016/j.injury.2017.01.038 (2017).*	**DEAD**	Walking: NO	Non-ambulatory	Non-ambulatory
		Breathing: NO	Not breathing	Not breathing
	**PRIORITY 1**	Walking: NO	Non-ambulatory	Non-ambulatory
		Breathing: YES	Breathing	Breathing/open airway
		Respiratory Rate: <12 or ≥22	RR≥22/min or RR<12/min	RR<br>_≥22/min or <12/min
		Heart Rate: ≥100	HR ≥100/min	HR<br>_≥100/min
		Glascow Coma Scale <14: YES	GCS <14	Following commands/neurological status<br>_GCS <14
	**PRIORITY 2**	Walking: NO	Non-ambulatory	Non-ambulatory and not [black (PX)] or [red (P1)]
		Respiratory Rate: ≥12 and <22	Not fulfilling any [black] or [red] criteria	
		Heart Rate: <100		
		Glascow Coma Scale <14: NO		
	**PRIORITY 3**	Walking: YES	Ambulatory	Ambulatory
**ASAV**<br><br>*Wolf, P., Bigalke, M., Graf, B. M., Birkholz, T. & Dittmar, M. S. Evaluation of a novel algorithm for primary mass casualty triage by paramedics in a physician manned EMS system: a dummy based trial. Scand J Trauma Resusc Emerg Med 22, 50, doi:10.1186/s13049-014-0050-6 (2014).*	**DEAD**	Ambulating: NO	Non-ambulatory	Non-ambulatory
		Deadly injured: YES	Deadly injured	Obvious signs of death
	**PRIORITY 1**	Ambulating: NO	Non-ambulatory	Non-ambulatory
		Deadly injured: NO	Not deadly injured	Not deadly injured
		Breathing difficulties: YES (Keep airway open!)<br>(Definition:<br>- Airway obstructed<br>- Bradypnoea, apnoea<br>- Dyspnoea, tachypnoea (not obviously psychogenic)<br>- Cyanosis)	Breathing difficulties (defined)	Respiratory distress
		Spurting hemmorhage: YES<br>_Stop the bleeding! Successful: NO	Spurting hemmorhage persistent after attempt to control	Major hemmorhage persistent after attempt to control
		Radial pulse absent: Pulse absent	Radial pulse absent	Radial/peripheral pulse absent
		Unable to follow simple commands: Unable	Unable to follow commands	Following commands/neurological status<br>_unable
	**PRIORITY 2**	Ambulating: NO	Non-ambulatory	Non-ambulatory and not [black (PX)] or [red (P1)]
		Deadly injured: NO	Not fulfilling any [black] or [red] criteria	
		Breathing difficulties: NO		
		Spurting hemmorhage: YES/NO<br>_Stop the bleeding! Successful: YES		
		Radial pulse absent: palpable		
		Unable to follow simple commands: Follows commands		
	**PRIORITY 3**	Ambulating: YES	Ambulatory	Ambulatory

APPX5_PHRASE_REPHRASE_MERGE

**SALT**<br><br>*Lerner, E. B. et al. Mass casualty triage: an evaluation of the science and refinement of a national guideline. Disaster Med Public Health Prep 5, 129-137, doi:10.1001 /dmp.2011.39 (2011).*	**DEAD**	(After performing LSI if needed)		
		Breathing: NO	Not breathing after:<br>- Positioning of airway and/or;<br>- Chest decompression	Not breathing<br>_after 1-2 attempts at positioning airway<br>_after chest decompression
	**IMMEDIATE**	Likely to survive given current resources: YES	Likely to survive given current resources	Likely to survive given current resources
		Breathing: YES	Breathing	Breathing/open airway
		Obeys commands or makes purposeful movements: NO	Not following commands or making purposeful movements	Following commands/neurological status<br>_unable<br>__...or not making purposeful movements
		Has peripheral pulse: NO	Peripheral pulse absent	Radial/peripheral pulse absent
		Not in respiratory distress: NO	In respiratory distress	Respiratory distress
		Major hemmorhage is controlled: NO	Major hemorrhage persistent after attempt to control	Major hemorrhage persistent after attempt to control
	**DELAYED**	Breathing: YES	Not fulfilling any [black (PX)] or [red (P1)] criteria	Not fulfilling any [black (PX)] or [red (P1)] criteria
		Obeys commands or makes purposeful movements: YES		
		Has peripheral pulse: YES		
		Not in respiratory distress: YES		
		Major hemmorhage is controlled: YES		
		Minor injuries only: NO	More than minor injuries	More than minor injuries
	**MINOR**	Breathing: YES	Not fulfilling any [black] or [red] criteria	Not fulfilling any [black] or [red] criteria
		Obeys commands or makes purposeful movements: YES		
		Has peripheral pulse: YES		
		Not in respiratory distress: YES		
		Major hemmorhage is controlled: YES		
		Minor injuries only: YES	Only minor injuries	Only minor injuries
	**EXPECTANT**	Likely to survive given current resources: NO	EXPECTANT/GRAY<br>- Not following commands or make purposeful movements and/or;<br>- Peripheral pulse absent and/or;<br>- In respiratory distress and/or;<br>- Major hemorrhage persistent after attempt to control<br>*...and **not likely to survive given current resources***	EXPECTANT/GRAY<br>- Not following commands or make purposeful movements and/or;<br>- Peripheral pulse absent and/or;<br>- In respiratory distress and/or;<br>- Major hemorrhage persistent after attempt to control<br>*...and **not likely to survive given current resources***
		Breathing: YES		
		Obeys commands or makes purposeful movements: NO		
		Has peripheral pulse: NO		
		Not in respiratory distress: NO		
		Major hemmorhage is controlled: NO		
**CFT**<br><br>*Garner, A., Lee, A., Harrison, K. & Schultz, C. H. Comparative analysis of multiple-casualty incident triage algorithms. Ann Emerg Med 38, 541-548, doi:10.1067/mem.2001.119053 (2001).*	**UNSALVAGEABLE**	Walks: NO	Non-ambulatory	Non-ambulatory
		Obeys command: NO	Not following commands	Following commands/neurological status<br>_unable
		Breathes with open airway: NO	Not breathing with an open airway	Not breathing
	**IMMEDIATE**	Walks: NO	Non-ambulatory	Non-ambulatory
		Obeys command: NO	Not following commands	Following commands/neurological status<br>_unable
		Breathes with open airway: YES	Breathing with an open airway	Breathing/open airway
		Palpable radial pulse: NO	No palpable radial pulse	Radial/peripheral pulse absent
	**URGENT**	Walks: NO	Non-ambulatory	Non-ambulatory and not [black (PX)] or [red (P1)]
		Obeys command: YES	Not fulfilling any [black] or [red] criteria	
		Palpable radial pulse: YES		
	**DELAYED**	Walks: YES	Ambulatory	Ambulatory
**TS**<br><br>*Garner, A., Lee, A., Harrison, K. & Schultz, C. H. Comparative analysis of multiple-casualty incident triage algorithms. Ann Emerg Med 38, 541-548, doi:10.1067/mem.2001.119053 (2001).*	**DEAD**	Walking: NO	Non-ambulatory	Non-ambulatory
		Airway: NO	Airway not open	Not breathing
	**IMMEDIATE**	Walking: NO	Non-ambulatory	Non-ambulatory
		Airway: YES	Open airway	Breathing/open airway
		Respiratory rate: Below 10 or over 29	RR>29/min or RR<10/min	RR<br>_>29/min or <10/min
		Capillary refill: Over 2s	CF >2 sec	CF >2 sec
		Heart rate: Over 120/min	HR >120/min	HR<br>_>120/min
	**URGENT**	Walking: NO	Non-ambulatory	Non-ambulatory and not [black (PX)] or [red (P1)]
		Airway: YES	Not fulfilling any [black] or [red] criteria	
		Respiratory rate: 10-29		
		Capillary refill: Under 2s		
		Heart rate: Under 120/min		
	**DELAYED**	Walking: YES	Ambulatory	Ambulatory
